# Exploring the role of expectations and stimulus relevance on stimulus-specific neural representations and conscious report

**DOI:** 10.1093/nc/niz011

**Published:** 2019-08-21

**Authors:** Erik L Meijs, Pim Mostert, Heleen A Slagter, Floris P de Lange, Simon van Gaal

**Affiliations:** 1 Radboud University Medical Center, Donders Institute for Brain, Cognition and Behaviour, Nijmegen 6500 HB, the Netherlands; 2 Donders Institute for Brain, Cognition and Behaviour, Radboud University, Nijmegen 6500 HB, the Netherlands; 3 Department of Psychology, University of Amsterdam, Amsterdam 1001 NK, the Netherlands; 4 Amsterdam Brain and Cognition (ABC), University of Amsterdam, Amsterdam 1001 NK, the Netherlands

**Keywords:** attentional blink, consciousness, magnetoencephalography, expectation, visual perception, decoding

## Abstract

Subjective experience can be influenced by top-down factors, such as expectations and stimulus relevance. Recently, it has been shown that expectations can enhance the likelihood that a stimulus is consciously reported, but the neural mechanisms supporting this enhancement are still unclear. We manipulated stimulus expectations within the attentional blink (AB) paradigm using letters and combined visual psychophysics with magnetoencephalographic (MEG) recordings to investigate whether prior expectations may enhance conscious access by sharpening stimulus-specific neural representations. We further explored how stimulus-specific neural activity patterns are affected by the factors expectation, stimulus relevance and conscious report. First, we show that valid expectations about the identity of an upcoming stimulus increase the likelihood that it is consciously reported. Second, using a series of multivariate decoding analyses, we show that the identity of letters presented in and out of the AB can be reliably decoded from MEG data. Third, we show that early sensory stimulus-specific neural representations are similar for reported and missed target letters in the AB task (active report required) and an oddball task in which the letter was clearly presented but its identity was task-irrelevant. However, later sustained and stable stimulus-specific representations were uniquely observed when target letters were consciously reported (decision-dependent signal). Fourth, we show that global pre-stimulus neural activity biased perceptual decisions for a ‘seen’ response. Fifth and last, no evidence was obtained for the sharpening of sensory representations by top-down expectations. We discuss these findings in light of emerging models of perception and conscious report highlighting the role of expectations and stimulus relevance.

## Introduction

What we perceive can be strongly influenced by top-down factors, such as our prior expectations about likely states of the world and the relevance of input for the task at hand (Bar 2004; Friston 2005; Bar *et al.* 2006; Clark 2013). According to a growing body of work, expectations, originating from past experience, can shape perception on both a neural and behavioural level (Summerfield and De Lange 2014). When sensory input matches prior expectations, performance on tasks is higher (Cheadle *et al.* 2015; Stein and Peelen 2015) and neural activity is attenuated (Alink *et al.* 2010; Kok *et al.* 2012; Todorovic and de Lange 2012). In such predictive brain frameworks, it is assumed that what we perceive consciously is strongly related to the brain’s best guess about the current state of the outside world (Gregory 1980; Hohwy 2012; Panichello *et al.* 2013; King and Dehaene 2014a).

Indeed, the idea that our subjective experience is strongly influenced by top-down factors is supported by numerous behavioural studies that have shown beneficial effects of prior knowledge on subjective perception, such as on the accuracy (Stein and Peelen 2015) or speed (Chang *et al.* 2015; Pinto *et al.* 2015) of detecting a stimulus. Likewise, it has been shown that prior knowledge increases the likelihood that a stimulus is consciously reported during the attentional blink (AB) (Martens and Johnson 2005; Visser *et al.* 2015; Meijs *et al.* 2018). Although expectations seem to affect conscious access, the neural underpinnings of these expectation-related modulations are still unclear. In a recent electroencephalographic (EEG) study, we did not find evidence that the amplitude of neural signals, as indexed by event-related potentials, explained the effect of expectations on the likelihood of conscious report of the stimulus (Meijs *et al.* 2018). This finding may be explained by the fact that instead of modulating the strength of neural responses, expectations may improve the signal-to-noise ratio or sharpness of the representation of stimuli (Kok *et al.* 2012; Bell *et al.* 2016) by instantiating specific perceptual templates (e.g. orientation selectivity), even before stimulus presentation (Kok *et al.* 2017). Because it has been shown previously that conscious perception may also be closely linked to the quality or variability of sensory representations (Schurger *et al.* 2010; *et al.*^2015^), we here investigated whether similar enhancements of neural representations also underlie the effects that expectations have on the conscious accessibility of stimuli (Meijs *et al.* 2018).

**Figure 1.**
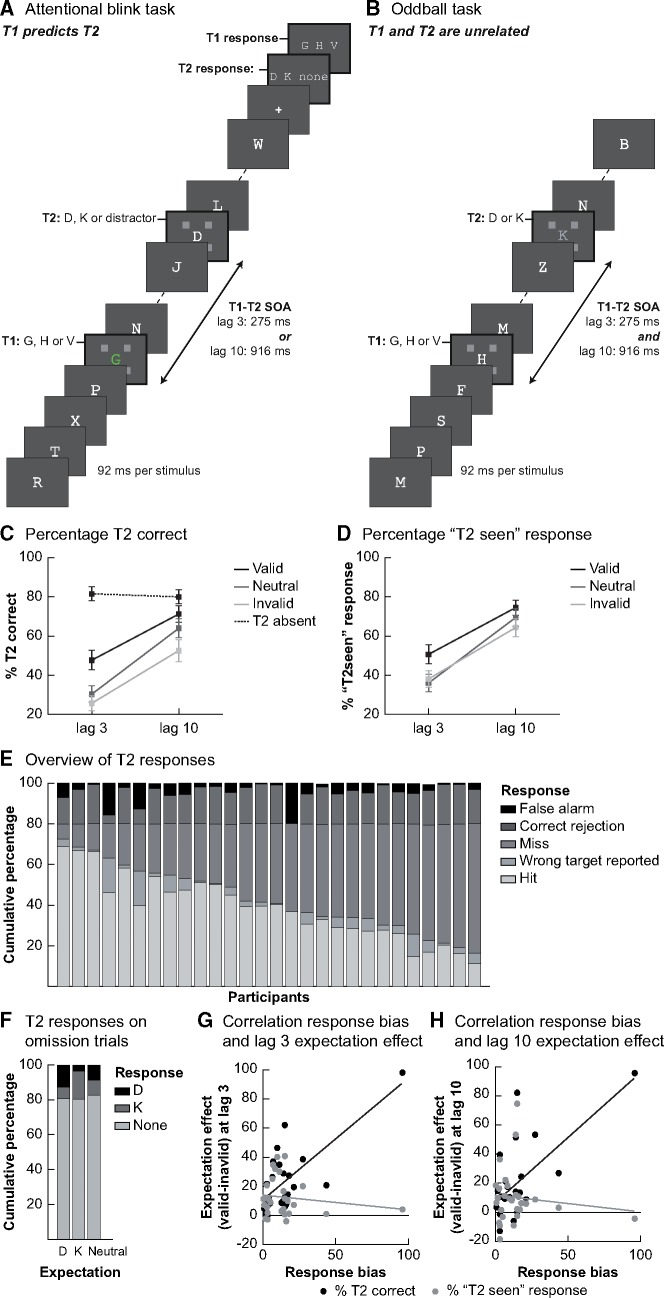
Experimental tasks and behavioural results. (**A**) The trial structure of the AB task. Each trial consisted of a sequence of rapidly presented letters in which targets were to be detected and reported at the end of the stream. Targets were marked by placeholders. The first target (T1: G, H or V) was always the fifth stimulus in the sequence. A second target (T2: D or K) was presented on 80% of trials, at varying lags. In a training session participants learned conditional probabilities between T1 and T2. One T1 stimulus was used as neutral condition and was thus followed equally often by each T2 stimulus. The other T1 stimuli predicted which T2 target was most likely to appear, thereby introducing valid and invalid expectations. (**B**) The oddball task was designed such that temporally it resembled the AB task as much as possible, with the most notable difference that on every trial three target stimuli were presented (T1, T2 at lag 3 and T2 at lag 10). Participants’ task was unrelated to the target identity: they had to respond to oddball stimuli that were present on 10% of trials at one of the target positions. In the example trial here, an oddball is presented at the T2 time point. (**C**) Percentage correct T2 (given that T1 was correctly identified) for each of the lags and conditions. Expectation validity significantly modulated the percentage of T2s that was consciously seen and this effect was different at the two lags. At lag 3, the expectation effect was mainly driven by the valid condition, while at lag 10 both invalid and valid expectations affected T2 detection as compared to neutral trials. (**D**) Results from a control analysis, using the percentage of trials on which participants reported seeing a T2 target (given that T1 was correctly identified) as dependent variable. Overall, the pattern of results was similar to that in (C). Error bars denote SEM. (**E**) A general overview of the response pattern over all trials for both lags together. The percentage of misses (no target reported when one was presented) was high in all participants. When no target was presented, participants usually correctly reported not seeing one. (**F**) The response pattern shows participants had a response bias: if they reported a target on a T2-absent trial, they more often guessed the expected target letter (when there was an expectation). (**G**) and (**H**) The relationship between the response bias (% of T2-absent trials with a predictive T1 where the expected target was reported) and the behavioural effect of expectation validity (valid–invalid) at lag 3 (**G**) and lag 10 (**H**) was depicted. In both, the relationship is shown for both outcome measures that were used to quantify behavioural performance: percentage correct (black) and percentage of ‘T2 seen’ responses (regardless of the exact letter participants entered; grey).

To this end, we used an AB task in which each trial consisted of a sequence of rapidly presented letters in which one or two targets were to be detected and reported at the end of the stream (targets were marked by placeholders, Fig. 1A). Crucially, the first target stimulus (T1) could either validly, invalidly or not predict (neutral trials) the identity of the second target (T2). Then, multivariate decoding analyses were used on magnetoencephalography (MEG) data to track the neural representations of target stimuli. Further, to explore the effects of task relevance on stimulus-specific neural activity patterns subjects also performed an additional ‘oddball’ task in which a similar rapid serial presentations of letters was presented but subjects were instructed to merely detect a contrast change of a stimulus that happened only on 10% of the trials (these oddball trials were not taken into account in the analyses). The combination of both tasks allowed us to trace the sensory processing of a letter stimulus in the absence of a target identity decision (because the identity of the letters was task-irrelevant). This decoding profile could then be compared to the decoding profile that we observed during the AB task in which subjects did have to make a perceptual decision on the presented target stimuli (target identity was task-relevant). This approach allowed us to perform within-task decoding analyses (training and testing on the AB task using k-folding) and between-task decoding analyses (training on the oddball task and testing on the AB task). Both approaches are aimed at testing different hypotheses. Training the classifier on the oddball task will extract a relatively pure sensory signal (letter identity is task-irrelevant) and therefore between-task decoding will likely isolate those neural processes in the AB task related to similar sensory stages of information processing. Based on earlier research (Kok *et al.* 2012, 2017), it may be predicted that these stages are also associated with the effect of expectations on conscious report. On the other hand, within-task decoding in the AB will train and test on task-relevant stimuli that require a categorical decision and therefore may additionally reveal decision-dependent processes that could also underlie the role of expectation in conscious access. Thus, our approach allowed us to examine if expectations influence visual representations at sensory and/or decision-related stages and how this influences conscious access.

## Materials and Methods

### Participants

We tested a total of 33 participants for this experiment. All participants had normal or corrected-to-normal vision. One participant was excluded because T1 identification performance was more than three standard deviations lower than the group average. Furthermore, four participants were excluded because their subjective estimates of T2 visibility were unreliable. When they indicated they perceived a target they were not able to identify the target correctly more often than chance-level (*P* > 0.05 in a binomial test). Additionally, only in the MEG analyses we excluded participants for whom the number of observations in any of the relevant conditions (usually in the invalid condition, which had less trials) was lower than 10, similar to Meijs *et al.* (2018). As a result, we included 28 participants (18 females, age 22.5 ± 2.8 years) in the behavioural analyses and 19 participants (12 females, age 23.0 ± 2.8 years) in the MEG analyses. For one control analysis, we included all 28 participants to rule out our selection of participant biased the results.

The experiment was approved by the local ethics committee of the Radboud University (CMO Arnhem-Nijmegen; ‘Imaging Human Cognition’). Written informed consent was obtained from participants according to the Declaration of Helsinki. Compensation was either 36 Euros or course credit.

### Materials

Stimuli were generated using the Psychophysics Toolbox (Brainard 1997) in a MATLAB (MathWorks, Natick, MA, USA) environment. In the behavioural lab, stimuli were displayed on a 24″ BENQ LED monitor (1920 × 1080 pixels; 120 Hz). A chinrest was used to control participants’ distance from the screen (±57 cm). In the MEG environment, a PROPixx projector (VPixx Technologies Inc., Saint-Bruno, Canada) located outside the magnetically shielded room projected the stimuli onto a screen ∼80 cm in front of participants (1920 × 1080 pixels, 120 Hz). All visual input was presented on a ‘black’ background (luminance: ± 3 cd/m^2^) and matched for visual luminance between the two labs.

### Procedure and stimuli

The experiment consisted of two sessions that were completed within 1 week. In the first session, participants performed an oddball task and in the second session an AB task (Raymond *et al.* 1992). In both sessions MEG was recorded. In the first session, after the oddball task, participants also practiced the AB task outside of the MEG scanner so that they could learn about the predictive relationship between T1 and T2.

#### Session 1: Oddball task

At the start of the first session, we measured MEG while participants performed an oddball task that was highly similar to the AB task (Fig. 1B). Again, every trial consisted of a sequence of letters in which letters that were targets in the AB task were presented. On every trial, a ‘T1’ (the letter D, V or G) was presented followed by a ‘T2’ (the letter D or K) at both lag 3 and lag 10 and every of these targets was marked by placeholders. All stimuli, including targets, were ‘white’. Every combination of ‘T1’ and ‘T2’ stimuli was equally likely, meaning there was no predictive relationship between the targets. Importantly, participants were not made aware of the presence of the targets. Moreover, because they had not yet seen the AB task, they did not know about the existence of any specific target letters (or predictive relationship between those). The timing of the stimuli and trials was identical to that in the AB task, with the exception that at the end of the letter sequence the ITI (800–1200 ms) started immediately.

Participants were instructed to detect oddball stimuli that occurred on 10% of the trials. An oddball was defined by its grey (luminance: ± 78 cd/m^2^) instead of white colour. Once an oddball was detected, the participant had to press the index finger button on an MEG-compatible button box as quickly as possible while the task continued. Because, unbeknownst to participants, oddballs were always either a ‘T1’ or ‘T2’ target, they were accompanied by the same placeholders used in the AB task. To make sure participants would focus their attention on the relevant time points in the sequence, they were explicitly instructed that these squares marked the potential temporal positions of oddballs and would help them detect oddballs. Oddball trials were excluded from all analyses. Every participant completed 8 blocks of 96 trials (total 768 trials) of the task. Every block was followed by summary feedback and a short break.

#### Session 1: Training of the AB task

At the end of the first session, every participant was behaviourally trained on the AB task. First, participants received on-screen instructions in which they were explicitly instructed about the predictive relationship between T1 and T2. Subsequently, they performed 6 blocks of 75 trials (total 300 trials) of the task. The goal of this training session was to familiarize participant with the task before the MEG session and to teach participants the predictive relationship between T1 and T2. We did not analyse the data from this training session.

#### Session 2: AB task

Participants had to detect targets within a sequence of rapidly presented distractors (92 ms per stimulus). Each stimulus in the sequence was an uppercase letter that was presented at fixation in a monospaced font (‘Courier New’; letter size: ±2.08°). The first target (T1: G, H or V) was presented in ‘green’ at the fifth position of the sequence. In 80% of trials a second target (T2: D or K) was presented as well, either at lag 3 (275 ms after T1 onset; two-third of trials) or at lag 10 (917 ms after T1 onset; one-third of trials). Each distractor letter (all alphabet letters excluding the targets) was presented maximally once per trial. T2 targets and distractors were presented in ‘white’ (luminance: ± 230 cd/m^2^).

Crucially, the likelihood of each T2 target appearing was conditional on the identity of the T1 stimulus that was previously presented (Fig. 1A). For every participant, one of the T1 stimuli (e.g. G) predicted that ‘D’ was the most likely T2 target while another T1 (e.g. H) made the exact opposite prediction that ‘K’ was the most likely T2 target. If a T2 was presented, the likely T2 stimulus was shown on 75% of trials. A third T1 stimulus (e.g. V) had no predictive value (neutral condition; 20% of trials), i.e. both T2 stimuli were equally likely to follow T1. All possible mappings of T1 and T2 were used across participants in a counterbalanced fashion, but mappings were fixed within a given participant for the entire experiment. On 20% of trials no T2 stimulus was presented but a random distractor letter was presented instead at either lag 3 or lag 10. Both at the T1-timepoint and the T2-timepoint of a trial, even when a T2 target was omitted, a placeholder consisting of four grey squares (luminance: ± 50 cd/m^2^; size: 0.62°; midpoint of each square centred at 2.34° horizontally and vertically from fixation) was presented around the target letter. This placeholder provided timing information, cueing participants which time points were relevant in a trial and thereby helping them decide which targets they saw during a trial.

Following a 150 ms blank period at the end of the letter sequence, participants gave their responses with the use of a (MEG-compatible) button box, using the index, middle and ring finger of their right hand. First, they reported the T2 they had seen (3 response options: D, K or none). Subsequently, they were asked to make a forced-choice judgment about the T1 stimulus that was presented (3 response options: G, H or V). A long response timeout duration of 4 s was used, and participants were explicitly instructed to value accuracy over response speed. The inter-trial interval was 800–1200 ms.

Before starting the MEG recordings, participants were briefly reminded about the task instructions (given to them in a previous session, see next paragraph). Every participant completed ∼750 trials (average: 748.7 ± 26.8, minimum: 652; maximum: 803), with the exact number depending on the duration of MEG preparations and the number of breaks a participant needed. At the end of every block of 75 trials, participants received summary feedback about their performance and were provided with the opportunity to take a short break.

### Behavioural analyses

#### Oddball task

Like for the AB task, behavioural data were preprocessed with MATLAB. We computed hit rates (percentage of detected oddballs) and analysed these using JASP (Love *et al.* 2015). Specifically, we compared the hit rate between trials where the oddball was presented at the T1-timepoint, the lag 3 T2-timepoint or the lag 10 T2-timepoint.

#### AB task

Behavioural data were preprocessed with in-house MATLAB scripts and subsequently analysed using JASP software (Love *et al.* 2015). We focused on the effects of lag and expectation on percentage correct T2 visibility, given that the correct T1 target was reported. T2 responses were considered to be correct if a participant entered the target letter that was presented or reported not seeing a letter when none was presented on a T2 absent trials. Since expectations are undefined on T2 absent trials, these trials cannot be used in the main statistical analyses. T2 percentage correct was used in a 2 × 2 repeated measures ANOVA with the factors expectation validity (valid, invalid and neutral) and lag (lag 3, lag 10). Subsequently, we performed *posthoc t*-tests to directly compare the different levels of expectations within each of the lags.

### MEG measurements and preprocessing

Whole-head magnetoencephalographic (MEG) recordings were acquired (sampling rate 1200 Hz) using a 275-channel MEG system with axial gradiometers (VSM/CTF Systems, Coquitlam, BC, Canada), which was located in a magnetically shielded room. Four channels (MLC11, MLC32, MLF62 and MRF66) were disabled in all participants for technical reasons. Head position was monitored and corrected if required using three coils, placed on the nasion and on earplugs in both ears (Stolk *et al.* 2013). Importantly, at the start of the second MEG session an effort was made to reposition a participants’ head as much as possible in the same location as during the first session by using a template head position saved in the first MEG session. In addition to the MEG, three sets of electrodes (+ground) were used to measure the electrocardiogram and horizontal and vertical eye movements. Finally, an Eyelink 1000 eyetracker (SR Research, Ottawa, Canada; sampling rate 1000 Hz) was used to measure pupil dilation and vertical and horizontal eye movements.

For each session separately, we preprocessed the data with the FieldTrip toolbox for MATLAB (Oostenveld *et al.* 2011). Data were high-pass filtered at 0.01 Hz to remove slow signal drifts. Additionally, a set of notch filters was applied at 50, 100 and 150 Hz to remove line noise. Subsequently, we cut the data into epochs from −750 to 1500 ms relative to T1 stimulus onset. The data were visually inspected and trials and/or channels with artefacts were deleted (averages session 1: 7.0% of trials, 1.2 channels; averages session 2: 5.4% of trials, 1.1 channels). To remove noise originating from far away external sources, third-order gradient correction using the CTF reference sensors was applied. Independent component analysis was used to identify and remove data components related to eye blinks, eye movements or heartbeats. To get a reliable estimate of which components to delete, each of the components was correlated to the EEG and eyetracker channels. Following the independent component analysis, previously deleted channels were reconstructed using the average of neighbouring channels. Finally, all trials were baseline corrected on the interval 500 ms prior to T1 onset (corresponding to −775 to −275 ms prior to T2 onset).

### Decoding analyses and statistics

Prior to the decoding analyses, we applied a sliding window of 50 ms to average the data, thereby smoothing the data in the temporal domain and improving the signal-to-noise ratio. Subsequently, we performed the decoding analyses by using linear discriminant analysis (LDA) decoders with the activity from all MEG-channels as features. In short, as outcome measure, the LDA decoder calculates the distance from a decision boundary on a trial-by-trial basis (full analysis details are available in Mostert *et al.* 2015). This distance measure can be used as a quantitative measure of the evidence for a certain class in de decoder. In cases where a decoder was trained and tested on the same dataset, a 10-fold cross-validation procedure was implemented in which for each fold the LDA decoder was trained on 90% of the trials and tested on the remaining 10% of trials. To be able to look at the stability of neural representations over time, all decoders were trained at one-time point and then tested on all-time points, resulting in a temporal generalization matrix (time range −250 to 1500 ms relative to T1; see also King and Dehaene 2014a,b). Cluster-based permutation tests with 1000 permutations were used to find the significant positive or negative clusters within the temporal generalization matrices of interest (Maris and Oostenveld 2007). During each permutation, positive and negative clusters were identified independently (each using one-sided *P* = 0.025 level), and subsequently the *t*-values within a cluster were summed. We then took the maximum absolute *t*-value sum as the statistic for the permutation distribution to which observed cluster *t*-statistics would be compared.

Using these analyses methods, we initially decoded T2 target identity at lag 3 within each of the two tasks. Further decoding analyses were done between-tasks, training the LDA decoder on one task and testing it on the other task. For the main analyses, we used the decoding that was trained on the oddball task and tested it on the AB task data (only trials where T1 was correctly identified), separately for valid and invalid trials. We did not include the neutral condition here because the number of observations in this condition was low (only presented on 20% of trials). A similar analysis was done to compare T2 identity decoding between T2 reported and T2 missed trials. As a control, we repeated all these decoding analyses for T2 targets presented at lag 10, the results of which are reported in the Supplementary material.

Next, we used the decoder trained on the oddball task and tested it on the AB data (T1 correct trials) for T2-absent trials and trials with lag 10 together (in both conditions, no target stimulus was presented at lag 3). Instead of grouping trials based on T2 identity, we grouped trials based on the expected T2 stimulus so that we could investigate the neural representation of sensory expectations. In a final group of analyses, decoders were trained and tested on the AB task (lag 3 and T1 correct trials only), allowing us to investigate the main effects of expectation validity and T2 visibility within the AB task. In addition, we did within-task T2 decoding analyses separately for T2 reported and T2 miss trials. Finally, to get a better view at the decoding results when training and testing on the same time point, we extracted the diagonal from the temporal generalization matrices of a number of analyses. We performed a paired-samples *t*-tests to each time point of the data after T1 presentation (0–1500 ms) and subsequently applied false discovery rate (FDR) correction.

## Results

### Behavioural results: oddball task

In the oddball task, we computed the hit rate (percentage of detected oddballs) for each of the time points at which an oddball could be presented. The hit rate was significantly different between these time points (*F*_2,__54_ = 13.87, *P* < 0.001; T1 time: 72.49 ± 27.91%; T2 lag 3 time: 74.38 ± 25.74%; T2 lag 10 time: 60.27 ± 27.12%). Follow-up *t*-tests showed this effect was caused by a lower hit rate for oddballs presented at the latest position in the stream (T1 time vs. T2 lag 10 time: *t*_27_ = 3.364, *P* = 0.002; T2 lag 3 time vs. T2 lag 10 time: *t*_27_ = 5.065, *P* < 0.001), while there was no difference between the other two time points (T1 time vs. T2 lag 3 time: *t*_27_ = −0.897, *P* = 0.378).

### Behavioural results: attentional blink task

In Fig. 1C, we show percentage correct T2 discrimination for trials on which T1 was correctly identified, separately for short and long lags (T1 accuracy T2 at lag 3: 95.63%, SD = 3.68%; T1 accuracy T2 at lag 10: 95.69%, SD = 3.95%). We observed a clear AB, as indicated by a reduced T2 performance when the time between T1 and T2 was short compared to long (lag 3: 34.57%; lag 10: 62.58%; *F*_1,__27_ = 61.43, *P* < 0.001). Importantly, T2 stimuli were more often correctly reported when they were expected (collapsed across lag: expected: 59.44%, unexpected: 39.13%; *F*_2,__54_=17.37, *P* < 0.001) and this effect of expectations was different at lag 3 than at lag 10 (interaction lag × validity: *F*_2,__54_ = 5.97, *P* = 0.005).

To test whether valid expectations increase, or invalid expectations decrease, T2 reportability, we directly compared valid, invalid and neutral trials at each of the two lags. At lag 3, where T2 most often goes unreported, T2 discrimination performance was higher in the valid (black line; 47.67%) than in the neutral (dark grey line; 30.35%) and invalid (light grey line; 25.68%) conditions (valid–invalid: *t*_27_ = 5.49, *P* < 0.001; valid–neutral: *t*_27_ = 4.60, *P* < 0.001). In the invalid condition performance was lowest, although not significantly lower than performance in the neutral condition (neutral–invalid: *t*_27_ = 1.79, *P* = 0.084). At the long lag, performance was also higher in the valid than invalid condition (valid 71.21%; invalid 52.57%; valid–invalid: *t*_27_ = 3.88, *P* < 0.001) and performance in the neutral condition was in between the other conditions, though closer to the valid condition (neutral: 63.97%; valid–neutral: *t*_27_ = 2.04, *P* = 0.051; neutral–invalid: *t*_27_ = 3.18, *P* = 0.004). Taken together, these results replicate earlier findings that conscious access can be affected by expectations about stimulus likelihood and that these effects are largest at short lags, inside the AB (Meijs *et al.* 2018). Additionally, at this short lag performance was significantly improved when expectations were valid compared to neutral, while the negative effect of invalid expectations was relatively small. Indeed, the benefit of valid expectations was larger at lag 3 than at lag 10 (valid–neutral lag 3 vs. valid–neutral lag 10: *t*_27_ = 3.67, *P* = 0.001). This suggests that at short lags, when the T2 stimulus is most often missed, the observed expectation effects are likely mostly driven by a benefit from valid expectations, which leads to a higher likelihood of conscious access.

Overall, false-alarms were infrequent, on ∼19% of T2-absent trials subjects reported a target (Fig. 1E and F), correct rejections for T2-absent trials: lag 3: 81.52%; lag 10: 79.94% (Aru and Bachmann 2017). Further, if participants reported seeing a target on T2-absent trials, they most often reported the expected target (*t*_27_ = 2.52, *P* = 0.018), demonstrating that participants had indeed learned the predictive relationship between T1 and T2 and used this information for their decisions. Therefore, it may be that the behavioural effect of expectations we observed on T2-present trials is (partly) affected by this response bias. Indeed, a correlation between participants’ response bias, as measured by the percentage of trials they reported the expected letter vs. another letter on T2-absent trials, and their expectation effect, as measured by the difference in performance on valid minus invalid trials, was observed at lag 3 (Fig. 1G and H, black dots; lag 3: spearman *r* = 0.49, *P* = 0.008; lag 10: spearman *r *= 0.34, *P* = 0.078). However, a control analysis where we analysed the percentage of ‘T2 seen’ responses (classifying a response as ‘seen’ regardless of whether this letter was correctly reported) showed comparable effects of expectation (validity effect: *F*_2,__54_ = 6.06, *P* = 0.004; interaction lag × validity: *F*_2,__54_ = 5.13, *P* = 0.009, Fig. 1D). Therefore it seems unlikely that response bias can fully explain our behavioural results, because in this control analysis the dependent variable (seen vs. miss) is completely orthogonal to participants’ expectations (D vs. K), ensuring that expectations cannot bias a specific response option. This is further supported by the fact that this outcome measure did not correlate with the response bias participants had on T2-absent trials (Fig. 1G and H; grey dots; lag 3: spearman *r* = −0.09, *P* = 0.649; lag 10: spearman *r* = −0.11, *P* = 0.594). This suggests that the observed expectation effects are present over and above the effects of response bias (note that the pattern of correlations was similar when the outlier subject with a response bias of nearly 100% was removed from the data, lag 3: spearman *r* = −0.05, *P* = 0.822; lag 10: spearman *r* = −0.05, *P* = 0.807).

### Decoding of T2 identity is modulated by stimulus relevance

**Figure 2.**
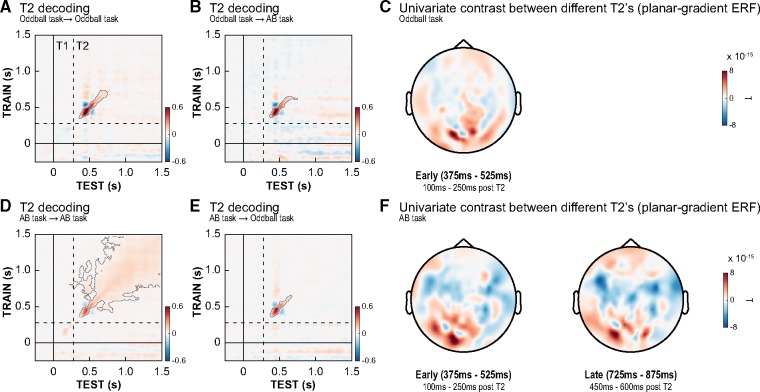
Decoding of T2 identity at lag 3. Temporal generalization matrices showing that T2 target identity (D vs. K) could be reliably decoded from MEG data within both the oddball task (**A**) and the AB task (**D**). Decoding was longer and more widespread in the latter, suggesting neural representations were more stable over time. (**B, E**) Between-task decoding analyses (trained and tested on separate datasets) showed significant clusters along the diagonal, indicating that early neural sensory representations of T2 identity were highly similar between-tasks. Timing of all panels is relative to T1 onset. Dashed lines indicate T2 stimulus onset. Contours of significant clusters are marked with a grey line (corrected for multiple comparisons). Decoding of T2 targets at lag 10 is shown in Supplementary Fig. 1. To visualize the pattern of activity used by the decoding algorithm, we display topographic plots (**C, F**) showing the planar gradient event related field (ERF)-difference between the two T2 targets (univariate contrast). On the left, we show the topography in an early window (100–250 ms post T2; including the peak in decoding) for both tasks. For the AB task only, a topographic plot in a later window (450–600 ms post T2), in which there was no significant decoding for the oddball task, is shown.

Multivariate analyses can provide insights in the dynamics of category-specific brain responses, as different neural and cognitive processes may be reflected in dissimilar patterns of temporal generalization (King and Dehaene 2014b). First, we tested the role of stimulus relevance in the processing of the letter stimuli. To do so, we focused on decoding the identity of T2 targets (D vs. K) at lag 3, irrespective of conscious report or expectations. Figure 2A and D shows the temporal generalization profiles for T2 identity within each of the two tasks, using cross-validation procedures and corrected for multiple comparisons (see Materials and Methods section for details). As can be seen, T2 target identity could reliably be decoded from MEG data in both tasks, as reflected in a typically observed diagonal decoding pattern starting ∼100 ms after T2 onset (Oddball task: *P* = 0.022; AB task: *P* < 0.001, permutation tests). This diagonal decoding pattern likely reflects a rapid sequence of distinct neural processes evolving over time (King and Dehaene 2014b; Mostert *et al.* 2015; Marti and Dehaene 2017). Further, as expected, in the AB task only, decoding performance was not only strong on the diagonal of the temporal generalization matrix, but its profile showed a mixture of a diagonal and a square-shaped pattern and decoding was much more extended in time. Thus, we observed stimulus-specific stable and sustained activation patterns for task-relevant compared to task-irrelevant stimuli. Next, we trained decoders on each of the tasks and subsequently tested them on the data of the other task (between-task decoding) to test for neural similarities and differences between the tasks. This approach solely resulted in significant clusters along the diagonal of the temporal generalization matrices for both analyses, further highlighting a shared neural coding of target identity from ∼100 to 375 ms post T2 onset (Oddball task → AB task: *P* = 0.013, Fig. 2B; AB task → Oddball task: *P* = 0.024, Fig. 2D). This conclusion is also supported by similar decoding results for T2 stimuli presented at lag 10 (Supplementary Fig. 1). Overall, these results show that when stimuli are task-relevant (require a perceptual decision) additional processing steps can be picked up by the classifier and T2 identity is processed in more stable and temporally extended neural representations, as compared to when stimuli are task-irrelevant (Kiebel *et al.* 2008; Stokes *et al.* 2009; King and Dehaene 2014b; Mostert *et al.*, 2015; Myers *et al.* 2017).

### Conscious access is related to late and stable stimulus-specific neural representations

**Figure 3.**
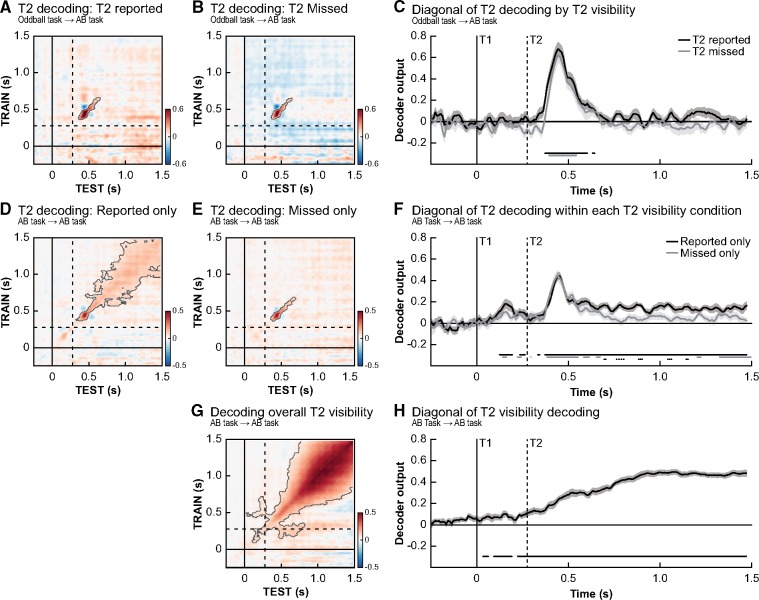
Conscious stimulus detection and its neural representation. Temporal generalization matrices for the decoding of T2 identity at lag 3, trained on the oddball task, for trials where T2 was consciously perceived (**A**) and trials where it was missed (**B**). T2 targets could be reliable decoded in both conditions and no significant differences between both conditions was observed along the diagonal (**C**). Within condition decoding analyses trained on the AB task show T2 decoding was more long-lasting (**F**) and stable on trials where T2 was consciously perceived (**D**) compared to where it was missed (**E**). (**G, H**) A direct investigation of the effect of T2 visibility (seen vs. miss, irrespective of stimulus identity) showed widespread significant decoding starting before stimulus onset, indicating an early and long-lasting main effect of stimulus visibility. Timing of all panels is relative to T1 onset and in every panel only trials where T1 was correctly identified were used. Dashed lines indicate the onset of a T2 stimulus at lag 3. Significant clusters in the temporal generalization matrices are contoured by a grey line (corrected for multiple comparisons). We also used *t*-test for effects along the diagonals, as shown in panels (C, F, H) in which significant time periods are marked by horizontal lines at the bottom of the panel. Solid lines indicate significant time points (FDR-correct *P*-value <0.05) for conditions while the difference between conditions is indicated by a dotted line (only in F).

Next, we tested how category-specific neural representations relate to conscious report, or the absence thereof, in the AB task. We did so by comparing T2 decoding (D vs. K) at lag 3 for seen T2s vs. missed T2s. To rule out any effect caused by the identity of T1, only trials where T1 was correctly reported were incorporated in these analyses. Figure 3 shows both between-task analyses (Oddball task → AB task) and within-task analyses (AB task → AB task; done separately per condition). In line with prevalent theoretical models of consciousness, such as global workspace theory (Baars 2002) and local recurrence theory (Lamme 2006), early sensory processing of the stimulus was independent of conscious report, as reflected by similar decoding profiles along the diagonal of the temporal generalization matrix for reported and missed T2’s (Fig. 3A–C, Oddball task → AB task) (Dehaene *et al.* 2006; Dehaene and Changeux 2011; King *et al.* 2016; Fahrenfort *et al.* 2017). Figure 3 also shows within-task analyses (AB task →AB task) in which T2 identity was decoded based on solely T2 reported (Fig. 3D) or T2 missed trials (Fig. 3E). In both analyses, significant l decoding of T2 identity around the diagonal was observed (T2 reported trials: *P* < 0.001, miss trials: *P* = 0.042). However, when T2 was reported, this decoding pattern was longer-lasting (Fig. 3F) and had a broader spatial profile (more square-shaped instead of diagonal, T2 reported: decoding significant from ∼100 ms to ∼1200 ms post T2-onset; T2 missed: decoding significant from ∼100 ms to ∼410 ms post T2-onset). This suggests that, compared to missed T2’s, the signal was broadcasted higher up the cortical hierarchy (for similar results see Mostert *et al.* 2015). Please note that the apparent pre-stimulus decoding in Fig. 3F should not necessarily be interpreted as evidence for decoding of stimulus-specific T2 activations before the stimulus is presented. This effect most likely relates to decoding of T1, which in the AB task was correlated to T2 identity.

Finally, we aimed to decode reported vs. missed T2’s in the AB task, irrespective of the specific stimulus that was presented (classifier labels: reported vs. missed, target letter is not relevant, Fig. 3G, *P* < 0.001). Several interesting aspects of the obtained temporal generalization profile can be noted. First, along the diagonal, decoding was significant already *before* T2 presentation (Fig. 3H). Neural activity before stimulus presentation thus predicted later stimulus report, in line with recent reports highlighting pre-stimulus fluctuations in neural activity that correlate with stimulus visibility (e.g. Linkenkaer-Hansen *et al.* 2004; Mathewson *et al.* 2009; Iemi *et al.* 2017). Because this analysis only included T1-correct trials, this pre-stimulus effect cannot easily be explained in terms of differences in T1-performance. Nevertheless, we cannot rule out that T1-related processes affected these results, since others have shown that the extent of T1 processing may be different for T2 blink and T2 no-blink trials (Ouimet and Jolicœur 2007; Slagter *et al.* 2017). The second interesting aspect about this overall visibility effects was that the square-shaped pattern was much stronger and clearer. This stimulus-independent profile is likely related to an amplified P3-like component on T2 reported trials compared to T2 missed trials, which is picked up by the classifier algorithm (see Supplementary Fig. 5 for ERF results) (Sergent *et al.* 2005; Meijs *et al.* 2018).

### Valid expectations do not enhance early sensory representations of stimulus identity

**Figure 4.**
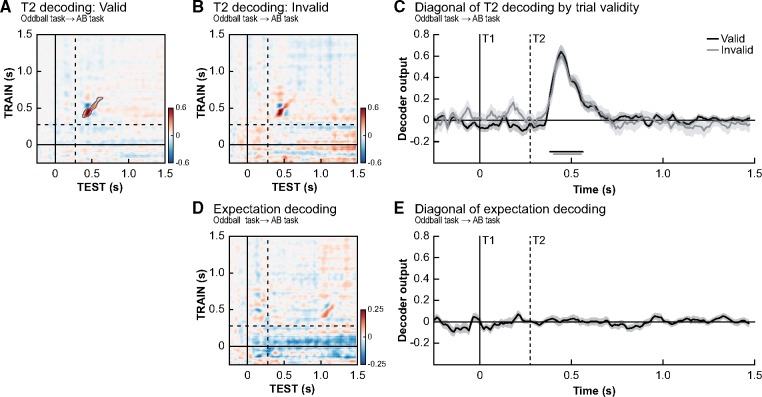
Expectation validity. Temporal generalization matrices for the decoding of T2 identity at lag 3 for valid trials (**A**) and invalid trials (**B**) separately. Only trials where T1 was correctly identified were used. (**C**) No significant differences (valid − invalid) between the two conditions were observed, indicating T2 identity could be decoded equally well on valid and invalid trials (significant time periods indicated by horizontal lines at bottom of panels, FDR-correct *P*-value <0.05), thus suggesting early sensory representations were not affected by the validity of prior expectations. (**D**, **E**) It was not possible to decode participant’s T2 stimulus expectations based on a between-task decoder trained on the oddball task data and tested on the attentional blink data. Timing is relative to T1 onset, and the dashed lines indicate the onset of T2 stimuli at lag 3. We did not find any evidence for the existence of neural stimulus templates.

In our final set of decoding analyses, we aimed to explore the link between neural recordings, expectations and conscious reports. Behaviourally, we observed that valid expectations increased the chance that a stimulus was subsequently reported and to test whether this may relate to the sharpening of sensory representation of T2 identity, decoding performance was compared between trials where participants had a valid (Fig. 4A) or invalid (Fig. 4B) expectation at lag 3 (only T1-correct trials were included). Importantly, only between-task analyses were performed in which the decoder was trained on the oddball task (no T1–T2 predictability) to prevent the decoding being biased by the predictive relationship between T1 and T2 in the first place, which is inherently present in our AB task and difficult to circumvent. Training on the AB task would also incorporate undesirable factors (e.g. conscious access: valid trials are reported more often than invalid trials) into the classifier training.

These decoding analyses revealed no effect of prior knowledge on the sharpening of sensory representations (T2 identity decoding was similar for valid and invalid trials, Fig. 4C, *P* > 0.9). Because several studies have shown that the effects of expectation may interact with (or even depend on) attention (Kok *et al.* 2012; Jiang *et al.* 2013), we aimed to rule out that the absence of expectation-related sharpening could be attributed to a lack of attention for targets presented at lag 3 (i.e. during the ‘blink’ period). However, similar results were obtained for stimuli presented at lag 10 and hence out of the AB period (Supplementary Fig. 3). Furthermore, we observed the same pattern of results in a control analysis with all the 28 participants that were included in the behavioural analyses. This eliminates the possibility that any absence of a significant effect may be related to the selection of participants (Supplementary Fig. 4). To verify whether there were any general neural effects of expectation validity, we also decoded whether a trial was valid or invalid regardless of T2 identity within the AB task (training and testing within the AB task). This control analysis, while matching the number of seen/unseen trials within each condition (valid vs. invalid), did not reveal significant decoding of validity (all cluster *P*-values >0.46). A final control analysis in which we trained the classifier on the difference between oddball stimuli and standard stimuli in the oddball task and tested the difference between valid and invalid trials in the AB task did also not reveal any effects of expectation violation (across task classification), but did reveal expectation violation signals in the oddball task itself (see Supplementary Fig. 6A).

The lack of a decoding difference between valid and invalid trials makes it unlikely that participants’ expectations induced neural ‘perceptual templates’ in the current task design. On valid trials, when participants’ expectation about the upcoming T2 was equal to the T2 that was presented, a decoder trained to distinguish T2 identity in the oddball task should have been able to pick up the representation of the target (expectation) before target presentation (target and expectation matched). Similarly, on invalid trials one would have expected inverse decoding, because participant’s expectation was opposite to the T2 stimulus that was later presented (target and expectation mismatched; Kok *et al.* 2017). To fully rule out that we may have missed expectation-induced sharpening, we performed a final analysis in which we grouped trials based on the expected T2 stimulus instead of the actually presented T2 stimulus. Only T2-absent and lag 10 trials were used, so no T2 stimulus was ever presented at lag 3 in order to prevent any contamination of the results by actual stimuli. But again we did not observe the instigation of expectation-related perceptual templates (all *P* > 0.221, Fig. 4D and E).

## Discussion

In this report, we investigated the relationship between expectations and conscious access and the role of stimulus relevance on stimulus-specific neural representations. Using an AB paradigm in which the identity of T1 predicted the likelihood of the T2, we found that T2 stimuli confirming T1-induced expectations are more likely to be consciously reported than T2 stimuli that violate these expectations. This is in line with studies that have shown that the speed (Chang *et al.* 2015; Pinto *et al.* 2015; De Loof *et al.* 2016) and accuracy (Stein and Peelen 2015; Meijs *et al.* 2018) of stimulus detection is strongly influenced by expectations. While control analyses in our experiment ruled out that response biases fully explain our effect, future experiments may benefit from the use of other measures, e.g. by asking participants to rate the subjective strength of the targets, such as done with the Perceptual Awareness Scale (Overgaard *et al.* 2006). Interestingly, by including neutral expectation trials we showed that the effect of expectations was mainly driven by a performance benefit for valid expectations, at least in the AB interval (at short lags) (see also Pinto *et al.* 2015). Thus, at the time that targets are often missed, conscious access becomes more likely when expectations are confirmed (valid) compared to when they were absent or invalidated (Fig. 1C).

We have also presented a series of multivariate decoding analyses, in which we first showed that it was possible to reliably decode the identity of a target letter from MEG data, both when the identity of this letter was task-irrelevant in an oddball task and when it was task-relevant and actively searched for in an AB task. Furthermore, the combination of both within- and between-task decoding analyses revealed that stimulus-specific neural representations underlying the early stages of information processing (on-diagonal generalization) were highly similar for both tasks and independent of stimulus relevance and the outcome of the perceptual decision (whether T2’s were reported or missed in the AB task; Mostert *et al.* 2015; King *et al.* 2016). Similar early decoding profiles were observed for (i) task-irrelevant letters in the oddball task, (ii) task-relevant, but missed T2’s in the AB task and (iii) task-relevant and reported T2’s in the AB task. These results suggest that early stimulus-specific neural processing stages are not modulated by either attention to the stimulus dimension [stimulus (task) relevance] nor conscious access to the target identity (report), in line with previous findings (Fahrenfort *et al.* 2017). The absence of decoding at later time points when T2 was task-irrelevant furthermore suggests that the representation of target letter identity is not ‘broadcasted’ to higher levels of the cortical hierarchy when this feature is task-irrelevant (Marti and Dehaene 2017). Usually, it is assumed that compared to nonconscious perception, conscious report is related to widespread neural activity (up to frontal areas) and more stable neural representations (Lamme and Roelfsema 2000; Dehaene *et al.* 2006; Dehaene and Changeux 2011; Schurger *et al.* 2015). Indeed we observed that within the AB task, when targets were task-relevant, attended and reported, T2 identity could be decoded for a longer time frames and was more stable, reflected in a square-shaped decoding profile (Ress *et al.* 2000; Jehee *et al.* 2011; Mostert *et al.* 2015; King *et al.* 2016; Marti and Dehaene 2017; van Vugt *et al.* 2018). Additionally, we observed a non-specific (regardless of T2 identity or expectations) difference between T2 reported and missed trials, of which the late sustained part presumably corresponds to previously observed P3 modulations related to conscious report (Kranczioch *et al.* 2003; Sergent *et al.* 2005; Meijs *et al.* 2018) and task relevance (Pitts *et al.* 2014; *et al.*^2014^). We also observed that fluctuations in neural activity (or overall ‘brain state’) before T2 presentation influenced whether or not the stimulus was later reported, as has been recently demonstrated by several others using at threshold or backward masking tasks (Linkenkaer-Hansen *et al.* 2004; Busch *et al.* 2009; Mathewson *et al.* 2009; Pincham and Szucs 2012; de Gee *et al.* 2014; Petro and Keil 2015; Iemi *et al.* 2017). These fluctuations may reflect differences in T1 processing (Slagter *et al.* 2017).

Next, in order to explain the behavioural effects of expectations on conscious access, we looked at the neural representations of T2 identity conditioned on the validity of single-trial expectations. We hypothesized that the early neural representation of validly expected stimuli would be ‘sharpened’, and that this improvement in improved stimulus representation would relate to enhanced stimulus report (Kok *et al.* 2012; Cheadle *et al.* 2015). However, we did not observe this effect. Moreover, no evidence for expectation-induced pre-stimulus perceptual templates was observed, although these effects have been recently reported in a different task context (Kok *et al.* 2017). One possible explanation for this discrepancy in findings is that it may take longer than 300 ms to encode T1 and translate the T1-prediction into a sensory representation. Nevertheless, in our study, the influence of expectations on conscious report was not reflected in modulations of sensory representations prior to T2 presentation or at early stages in the processing hierarchy (for a similar conclusions in a different paradigm see e.g. Rungratsameetaweemana *et al.* 2018; Alilovic *et al.* 2019; Weaver *et al.* 2019). It could be that expectations can influence perception at multiple levels of the cortical hierarchy, with different processes being affected depending on the type of target stimuli or expectations being involved. In our AB paradigm, participants learned conditional relationships between two letter stimuli. Consequently, the expectations in our task were most likely represented at a higher level of the cortical hierarchy (i.e. semantic) as compared to the rather low-level perceptual expectations in earlier studies (Kok *et al.* 2012, 2017). As a consequence, the early sensory representation of the targets that we pick up based on the oddball task may not have been optimal for detecting expectation-based modulations of T2 processing. Therefore, the ‘format’ in which the expectations were represented may simply have been different (e.g. verbally or motor code) than that of the incoming visual information and hence interactive effects may not have been visible at the sensory level (Bang and Rahnev 2017; Rungratsameetaweemana *et al.* 2018; Rungratsameetaweemana and Serences 2019). Future research would benefit from using tasks that can capture the full stimulus processing hierarchy involved in processing the relevant stimuli within the experimental task (sensory, semantic, decision).

In line with a recent study (Rungratsameetaweemana *et al.* 2018), we did not find any reliable time-locked decoding effects that we could relate to the behavioural benefits of expectations, suggesting it may be relatively subtle and hard to detect (see also Meijs *et al.* 2018). Expectations may only affect small populations of neurons and such effect may be hard to detect using analyses based on data from multiple sources (which is the case here because analyses were performed on the sensor level). Such effects may be more readily picked up with analyses in the source domain. Alternatively, there are other possible implementations of expectations, such as the modulation of the onset of components related to conscious perception (Melloni *et al.* 2011). Other potentially interesting signatures are the power and/or phase of alpha oscillations because a number of studies have shown that low pre-stimulus alpha power is predictive of subsequent conscious stimulus perception (Linkenkaer-Hansen *et al.* 2004; Busch *et al.* 2009; Mathewson *et al.* 2009; de Lange *et al.* 2013; Sherman *et al.* 2016; Benwell *et al.* 2017; Iemi *et al.* 2017). Furthermore, it has been shown that expectations lead to changes in pre-stimulus alpha in a way that predicts stimulus visibility (Mayer *et al.* 2016). Future studies are required to unravel the effect of expectations on conscious access in the frequency domain.

A number of conclusions can be drawn from this study. First, we have shown that the neural representation of letters presented during rapid serial visual presentation can be reliably decoded from neural activity as measured by MEG. While early, sensory representations of letters could be decoded regardless of the behavioural state of the subject, later and more stable multivariate activity patterns were dependent on top-down modulations by task relevance, attention and conscious report. Second, we have shown that valid expectations enhance the likelihood of visual target detection but we did not find evidence that this was due to increased ‘sharpness’ of the relevant sensory representations.

### Data availability

The data and analysis scripts used in this article will be made publicly available after manuscript acceptance at the following web address http://hdl.handle.net/11633/di.dccn.DSC_3018015.05_065. Prior to accessing and downloading the shared data, users must create an account. It is possible to use an institutional account or a social ID from Google, Facebook, Twitter, LinkedIn or Microsoft. After authentication, users must accept the Data Use Agreement (DUA), after which they are automatically authorized to download the shared data. The DUA specifies whether there are any restrictions on how the data may be used. The Radboud University and the Donders Institute for Brain, Cognition and Behaviour will keep these shared data available for at least 10 years.

## Supplementary Material

niz011_Supplementary_material

## References

[niz011-B1] Alilovic J, Timmermans B, Reteig LC, et al No evidence that predictions and attention modulate the first feedforward sweep of cortical information processing. Cereb Cortex 2019;29:2261–78. 30877784 10.1093/cercor/bhz038 PMC6484894

[niz011-B2] Alink A, Schwiedrzik CM, Kohler A, et al Stimulus predictability reduces responses in primary visual cortex. J Neurosci 2010;30:2960–6. 20181593 10.1523/JNEUROSCI.3730-10.2010 PMC6633950

[niz011-B3] Aru J, Bachmann T. Expectation creates something out of nothing: the role of attention in iconic memory reconsidered. Conscious Cogn 2017;53:203–10. 28687418 10.1016/j.concog.2017.06.017

[niz011-B4] Baars BJ. The conscious access hypothesis: origins and recent evidence. Trends Cogn Sci 2002;6613:47–52. 10.1016/s1364-6613(00)01819-2 11849615

[niz011-B5] Bang JW, Rahnev D. Stimulus expectation alters decision criterion but not sensory signal in perceptual decision making. Sci Rep 2017;7:1–12. 29213117 10.1038/s41598-017-16885-2 PMC5719011

[niz011-B6] Bar M. Visual objects in context. Nat Rev Neurosci 2004;5:617–29. 15263892 10.1038/nrn1476

[niz011-B7] Bar M, Kassam KS, Ghuman AS, et al Top-down facilitation of visual recognition. Proc Natl Acad Sci USA 2006;103:449–54. 16407167 10.1073/pnas.0507062103 PMC1326160

[niz011-B8] Bell AH, Summerfield C, Morin EL, et al Encoding of stimulus probability in macaque inferior temporal cortex. Curr Biol 2016;26:2280–90. 27524483 10.1016/j.cub.2016.07.007 PMC5021632

[niz011-B9] Benwell CSY, Tagliabue CF, Veniero D, et al Prestimulus EEG power predicts conscious awareness but not objective visual performance. eNeuro 2017;4:ENEURO.0182-17.2017. 10.1523/ENEURO.0182-17.2017 PMC5732016 29255794

[niz011-B10] Brainard DH. The psychophysics toolbox. Spat Vis 1997;10:433–6. 9176952

[niz011-B11] Busch NA, Dubois J, VanRullen R. The phase of ongoing EEG oscillations predicts visual perception. J Neurosci 2009;29:7869–76. 19535598 10.1523/JNEUROSCI.0113-09.2009 PMC6665641

[niz011-B12] Chang AYC, Kanai R, Seth AK. Cross-modal prediction changes the timing of conscious access during the motion-induced blindness. Conscious Cogn 2015;31:139–47. 25486340 10.1016/j.concog.2014.11.005

[niz011-B13] Cheadle S, Egner T, Wyart V, et al Feature expectation heightens visual sensitivity during fine orientation discrimination. J Vis 2015;15:14. 10.1167/15.14.14 PMC4633117 26505967

[niz011-B14] Clark A. Whatever next? Predictive brains, situated agents, and the future of cognitive science. Behav Brain Sci 2013;36:181–204. 23663408 10.1017/S0140525X12000477

[niz011-B15] de Gee JW, Knapen T, Donner TH. Decision-related pupil dilation reflects upcoming choice and individual bias. Proc Natl Acad Sci USA 2014;111:E618–E625. 24449874 10.1073/pnas.1317557111 PMC3918830

[niz011-B16] de Lange FP, Rahnev DA, Donner TH, et al Prestimulus oscillatory activity over motor cortex reflects perceptual expectations. J Neurosci 2013;33:1400–10. 23345216 10.1523/JNEUROSCI.1094-12.2013 PMC6618755

[niz011-B17] De Loof E, Van Opstal F, Verguts T. Predictive information speeds up visual awareness in an individuation task by modulating threshold setting, not processing efficiency. Vision Res 2016;121:104–12. 26975499 10.1016/j.visres.2016.03.002

[niz011-B18] Dehaene S, Changeux J-P. Experimental and theoretical approaches to conscious processing. Neuron 2011;70:200–27. 21521609 10.1016/j.neuron.2011.03.018

[niz011-B19] Dehaene S, Changeux J-P, Naccache L, et al Conscious, preconscious, and subliminal processing: a testable taxonomy. Trends Cogn Sci 2006;10:204–11. 16603406 10.1016/j.tics.2006.03.007

[niz011-B20] Fahrenfort JJ, van Leeuwen J, Olivers CNL, et al Perceptual integration without conscious access. Proc Natl Acad Sci USA 2017;114:3744–9. 28325878 10.1073/pnas.1617268114 PMC5389292

[niz011-B21] Friston K. A theory of cortical responses. Philos Trans R Soc B 2005;360:815–36. 10.1098/rstb.2005.1622 PMC1569488 15937014

[niz011-B22] Gregory RL. Perceptions as hypotheses. Philos Trans R Soc B 1980;290:181–97. 10.1098/rstb.1980.0090 6106237

[niz011-B23] Hohwy J. Attention and conscious perception in the hypothesis testing brain. Front Psychol 2012;3:96. 22485102 10.3389/fpsyg.2012.00096 PMC3317264

[niz011-B24] Iemi L, Chaumon M, Crouzet SM, et al Spontaneous neural oscillations bias perception by modulating baseline excitability. J Neurosci 2017;37:807–19. 28123017 10.1523/JNEUROSCI.1432-16.2016 PMC6597018

[niz011-B25] Jehee JFM, Brady DK, Tong F. Attention improves encoding of task-relevant features in the human visual cortex. J Neurosci 2011;31:8210–9. 21632942 10.1523/JNEUROSCI.6153-09.2011 PMC3134176

[niz011-B26] Jiang J, Summerfield C, Egner T. Attention sharpens the distinction between expected and unexpected percepts in the visual brain. J Neurosci 2013;33:18438–47. 24259568 10.1523/JNEUROSCI.3308-13.2013 PMC3834051

[niz011-B27] Kiebel SJ, Daunizeau J, Friston KJ. A hierarchy of time-scales and the brain. PLoS Comput Biol 2008;4:e1000209. 10.1371/journal.pcbi.1000209 PMC2568860 19008936

[niz011-B28] King J-R, Dehaene S. A model of subjective report and objective discrimination as categorical decisions in a representational space. Philos Trans R Soc B2014a;369:20130204. 10.1098/rstb.2013.0204 PMC3965160 24639577

[niz011-B29] King J-R, Dehaene S. Characterizing the dynamics of mental representations: the temporal generalization method. Trends Cogn Sci2014b;18:203–210. 24593982 10.1016/j.tics.2014.01.002 PMC5635958

[niz011-B30] King J-R, Pescetelli N, Dehaene S. Brain mechanisms underlying the brief maintenance of seen and unseen sensory information. Neuron 2016;92:1122–1134. 27930903 10.1016/j.neuron.2016.10.051

[niz011-B31] Kok P, Jehee JFM, de Lange FP. Less is more: expectation sharpens representations in the primary visual cortex. Neuron 2012;75:265–270. 22841311 10.1016/j.neuron.2012.04.034

[niz011-B32] Kok P, Mostert P, de Lange FP. Prior expectations induce prestimulus sensory templates. Proc Natl Acad Sci USA 2017;114:201705652. 10.1073/pnas.1705652114 PMC5625909 28900010

[niz011-B33] Kok P, Rahnev D, Jehee JFM, et al Attention reverses the effect of prediction in silencing sensory signals. Cereb Cortex 2012;22:2197–2206. 22047964 10.1093/cercor/bhr310

[niz011-B34] Kranczioch C, Debener S, Engel AK. Event-related potential correlates of the attentional blink phenomenon. Cogn Brain Res 2003;17:177–187. 10.1016/s0926-6410(03)00092-2 12763203

[niz011-B35] Lamme VAF, Roelfsema PR. The distinct modes of vision offered by feedforward and recurrent processing. Trends Neurosci 2000;23:571–579. 11074267 10.1016/s0166-2236(00)01657-x

[niz011-B36] Lamme VAF. Towards a true neural stance on consciousness. Trends Cogn Sci 2006;10:494–501. 16997611 10.1016/j.tics.2006.09.001

[niz011-B37] Linkenkaer-Hansen K, Nikulin VV, Palva S, et al Prestimulus oscillations enhance psychophysical performance in humans. J Neurosci 2004;24:10186–10190. 15537890 10.1523/JNEUROSCI.2584-04.2004 PMC6730198

[niz011-B38] Love J, Selker R, Marsman M, et al (2015). *JASP* (Version 0.7) [computer software].

[niz011-B39] Maris E, Oostenveld R. Nonparametric statistical testing of EEG- and MEG-data. Journal of Neuroscience Methods 2007;164:177–190. 17517438 10.1016/j.jneumeth.2007.03.024

[niz011-B40] Martens S, Johnson A. Timing attention: cuing target onset interval attenuates the attentional blink. Memory Cogn 2005;33:234–240. 10.3758/bf03195312 16028578

[niz011-B41] Marti S, Dehaene S. Discrete and continuous mechanisms of temporal selection in rapid visual streams. Nat Commun 2017;8:1955. 10.1038/s41467-017-02079-x PMC5717232 29208892

[niz011-B42] Mathewson KE, Gratton G, Fabiani M, et al To see or not to see: prestimulus phase predicts visual awareness. J Neurosci 2009;29:2725–2732. 19261866 10.1523/JNEUROSCI.3963-08.2009 PMC2724892

[niz011-B43] Mayer A, Schwiedrzik CM, Wibral M, et al Expecting to see a letter: alpha oscillations as carriers of top-down sensory predictions. Cereb Cortex 2016;26:3146–3160. 26142463 10.1093/cercor/bhv146

[niz011-B44] Meijs EL, Slagter HA, de Lange FP, et al Dynamic Interactions between top–down expectations and conscious awareness. J Neurosci 2018;38:2318–2327. 29386259 10.1523/JNEUROSCI.1952-17.2017 PMC6596276

[niz011-B45] Melloni L, Schwiedrzik CM, Muller N, et al Expectations change the signatures and timing of electrophysiological correlates of perceptual awareness. J Neurosci 2011;31:1386–1396. 21273423 10.1523/JNEUROSCI.4570-10.2011 PMC6623627

[niz011-B46] Mostert P, Kok P, de Lange FP. Dissociating sensory from decision processes in human perceptual decision making. Sci Rep 2015;5:18253. 26666393 10.1038/srep18253 PMC4678878

[niz011-B47] Myers NE, Stokes MG, Nobre AC. Prioritizing information during working memory: beyond sustained internal attention. Trends Cogn Sci 2017;21:449–461. 28454719 10.1016/j.tics.2017.03.010 PMC7220802

[niz011-B48] Oostenveld R, Fries P, Maris E, et al FieldTrip: open source software for advanced analysis of MEG, EEG, and invasive electrophysiological data. Comput Intell Neurosci 2011;2011:156869. 10.1155/2011/156869 PMC3021840 21253357

[niz011-B49] Ouimet C, Jolicœur P. Beyond task 1 difficulty: the duration of T1 encoding modulates the attentional blink. Visual Cogn 2007;15:290–304.

[niz011-B50] Overgaard M, Rote J, Mouridsen K, et al Is conscious perception gradual or dichotomous? A comparison of report methodologies during a visual task. Conscious Cogn 2006;15:700–708. 16725347 10.1016/j.concog.2006.04.002

[niz011-B51] Panichello MF, Cheung OS, Bar M. Predictive feedback and conscious visual experience. Front Psychol 2013;3:620. 23346068 10.3389/fpsyg.2012.00620 PMC3549576

[niz011-B52] Petro NM, Keil A. Pre-target oscillatory brain activity and the attentional blink. Exp Brain Res 2015;233:3583–3595. 26341931 10.1007/s00221-015-4418-2 PMC4651748

[niz011-B53] Pincham HL, Szucs D. Conscious access is linked to ongoing brain state: electrophysiological evidence from the attentional blink. Cereb Cortex 2012;22:2346–2353. 22079924 10.1093/cercor/bhr314

[niz011-B54] Pinto Y, van Gaal S, de Lange FP, et al Expectations accelerate entry of visual stimuli into awareness. J Vis 2015;15:13. 10.1167/15.8.13 26114676

[niz011-B55] Pitts MA, Metzler S, Hillyard SA. Isolating neural correlates of conscious perception from neural correlates of reporting one’s perception. Front Psychol 2014;5:1–16. 25339922 10.3389/fpsyg.2014.01078 PMC4189413

[niz011-B56] Pitts MA, Padwal J, Fennelly D, et al Gamma band activity and the P3 reflect post-perceptual processes, not visual awareness. NeuroImage 2014;101:337–350. 25063731 10.1016/j.neuroimage.2014.07.024 PMC4169212

[niz011-B57] Raymond JE, Shapiro KL, Arnell KM. Temporary suppression of visual processing in an RSVP task: an attentional blink? J Exp Psychol 1992;18:849–860. Vol. 10.1037//0096-1523.18.3.849 1500880

[niz011-B58] Ress D, Backus BT, Heeger DJ. Activity in primary visual cortex predicts performance in a visual detection task. Nat Neurosci 2000;3:940–945. 10966626 10.1038/78856

[niz011-B59] Rungratsameetaweemana N, Itthipuripat S, Salazar A, et al Expectations do not alter early sensory processing during perceptual decision-making. J Neurosci 2018;38:5632–5648. 29773755 10.1523/JNEUROSCI.3638-17.2018 PMC8174137

[niz011-B60] Rungratsameetaweemana N, Serences JT. ScienceDirect dissociating the impact of attention and expectation on early sensory processing. Curr Opin Psychol 2019;29:181–186. 31022561 10.1016/j.copsyc.2019.03.014 PMC6756985

[niz011-B61] Schurger A, Pereira F, Treisman A, et al Reproducibility distinguishes conscious from nonconscious neural representations. Science 2010;327:97–99. 19965385 10.1126/science.1180029

[niz011-B62] Schurger A, Sarigiannidis I, Naccache L, et al Cortical activity is more stable when sensory stimuli are consciously perceived. Proc Natl Acad Sci USA 2015;112:E2083–E2092. 25847997 10.1073/pnas.1418730112 PMC4413285

[niz011-B63] Sergent C, Baillet S, Dehaene S. Timing of the brain events underlying access to consciousness during the attentional blink. Nat Neurosci 2005;8:1391–1400. 16158062 10.1038/nn1549

[niz011-B64] Sherman MT, Kanai R, Seth AK, et al Rhythmic influence of top–down perceptual priors in the phase of prestimulus occipital alpha oscillations. J Cogn Neurosci 2016;28:1318–1330. 27082046 10.1162/jocn_a_00973

[niz011-B65] Slagter HA, Mazaheri A, Reteig LC, et al Contributions of the ventral striatum to conscious perception: an intracranial EEG study of the attentional blink. J Neurosci 2017;37:1081–1089. 27986925 10.1523/JNEUROSCI.2282-16.2016 PMC6596850

[niz011-B66] Stein T, Peelen MV. Content-specific expectations enhance stimulus detectability by increasing perceptual sensitivity. J Exp Psychol 2015;144:1089–1104. 10.1037/xge0000109 26460783

[niz011-B67] Stokes M, Thompson R, Nobre AC, et al Shape-specific preparatory activity mediates attention to targets in human visual cortex. Proc Natl Acad Sci USA 2009;106:19569–19574. 19887644 10.1073/pnas.0905306106 PMC2772815

[niz011-B68] Stolk A, Todorovic A, Schoffelen JM, et al Online and offline tools for head movement compensation in MEG. NeuroImage 2013;68:39–48. 23246857 10.1016/j.neuroimage.2012.11.047

[niz011-B69] Summerfield C, De Lange FP. Expectation in perceptual decision making: neural and computational mechanisms. Nat Rev Neurosci 2014;15:745–756. 25315388 10.1038/nrn3838

[niz011-B70] Todorovic A, de Lange FP. Repetition suppression and expectation suppression are dissociable in time in early auditory evoked fields. J Neurosci 2012;32:13389–13395. 23015429 10.1523/JNEUROSCI.2227-12.2012 PMC6621367

[niz011-B71] van Vugt B, Dagnino B, Vartak D, et al The threshold for conscious report: signal loss and response bias in visual and frontal cortex. Science 2018;360:537–542. 29567809 10.1126/science.aar7186

[niz011-B72] Visser TW, Ohan JL, Enns JT. Temporal cues derived from statistical patterns can overcome resource limitations in the attentional blink. Attent Percept Psychophys 2015;77:1585–1595. 10.3758/s13414-015-0880-y 25813742

[niz011-B73] Weaver MD, Fahrenfort JJ, Belopolsky A, et al Independent neural activity patterns for sensory- and confidence-based information maintenance during category-selective visual processing. eNeuro 2019;6:e0268–18.2018 1–13. 10.1523/ENEURO.0268-18.2018 PMC6397950 30834301

