# Supplementary material for: Exploring the role of expectations and stimulus relevance on stimulus-specific neural representations and conscious report
Source: Neurosci Conscious. 2019 Aug 21;2019(1):niz011. doi: 10.1093/nc/niz011 (PMC6704346; doi:10.1093/nc/niz011)
Supplement: niz011_Supplementary_material [file niz011_supplementary_material.docx]

# Supplementary material

## Decoding results for T2 stimuli presented at lag 10

T2 identity for targets presented at lag 10 could be reliably decoded (**Supplementary Figure 1**) from MEG data both within-task (Oddball task: p=0.011; AB task: p=0.025) and between-task (Oddball task → AB task: p=0.025; AB task → Oddball task: p=0.020). This further validates the conclusion that the initial representation of T2 targets was similar between the tasks.


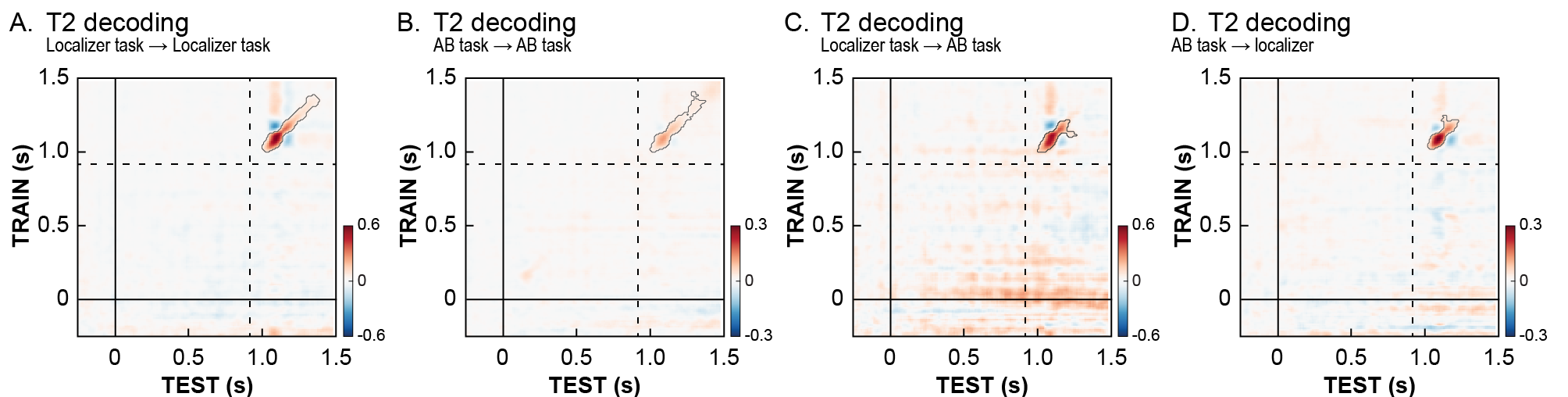


**Supplementary Figure 1. Decoding of T2 targets at lag 10.** We show the temporal generalization matrices for the decoding of T2 identity with different types of decoding analyses. Timing of all panels is relative to T1 onset. The dotted lines indicate the onset of T2 stimulus at lag 10. On the left, we show the decoding performance for cross-validated decoders that were trained and tested within either the oddball task **(A)** or attentional blink **(B)** task. On the right, we show the results for decoding analyses where the decoder was trained and tested on separate datasets. In **(C)** the decoder was trained on the oddball task and tested on the AB task, while in **(D)** the AB task was used for training and the oddball task was used for testing. The identity of T2 could be decoded in all the analyses. Contours of significant clusters are marked with a grey line (corrected for multiple comparisons).

The representation of T2 stimuli presented at lag 10 was similar for detected and missed T2s (**Supplementary Figure 2**; all p>0.0785). We should note that we did find effects for this contrast at lag 3, but these were on the off diagonal starting relatively late, making it likely they simply “fall outside” the time axis in the temporal generalization matrix.


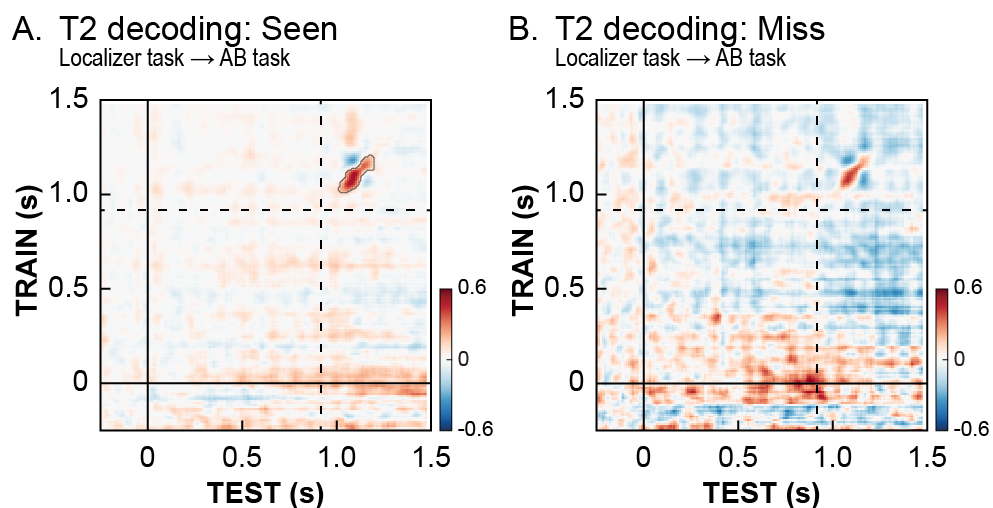


**Supplementary Figure 2. Effects of conscious stimulus detection on the neural representation of T2s at lag 10.** Temporal generalization matrices for the decoding of T2 identity at lag 10 for trials where the T2 was consciously perceived **(A)** and trials in which it was missed **(B)**. Only trials where T1 was correctly identified were used. The timing of all panels is relative to T1 onset. Dashed lines indicate the onset of a T2 stimulus at lag 10.

In line with the analyses performed for T2s presented at lag 3, no significant differences were observed between trials on which the stimulus was validly or invalidly expected (all p>0.582). These results show it is unlikely that the absence of an expectation validity effect at lag 3 was due to a lack of attention in early time-windows where attention “blinks” (Jiang et al., 2013).


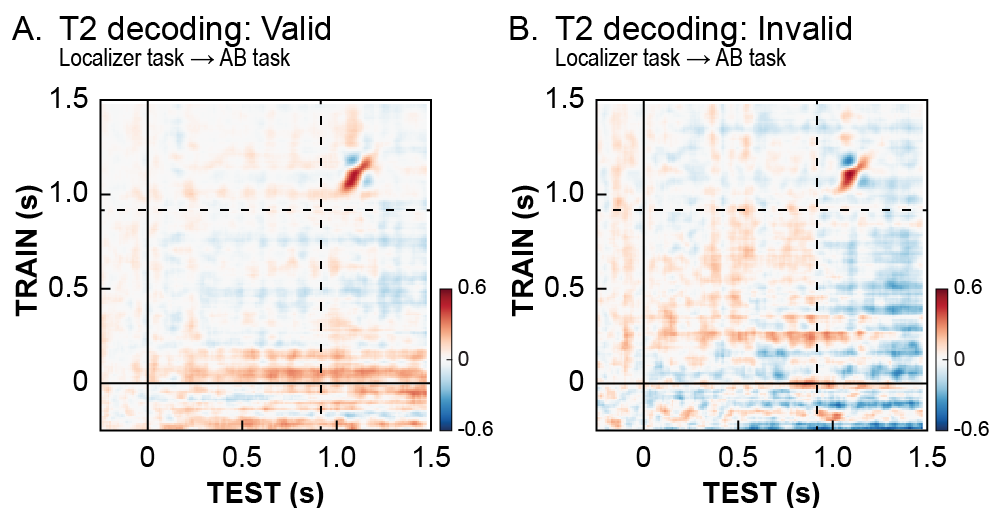


**Supplementary Figure 3. Effects of expectation validity on the neural representation of T2s at lag 10.** We show the temporal generalization matrices for the decoding of T2 identity at lag 10 for valid trials **(A)** and invalid trials **(B)** separately. Only trials where T1 was correctly identified were used. There were no significant differences (valid – invalid) between the two conditions. Timing of all panels is relative to T1 onset. Dashed lines indicate the onset of a T2 stimulus at lag 10.

To rule out the absence of decoding differences between valid and invalid trials at lag 3 was caused by our selection of participants, we replicated the analyses but not including all 28 participants who were included in the behavioural analyses. Again, no significant difference was observed between decoding T2 target identity in valid and invalid trials (**Supplementary Figure 4**, p>0.9).

**
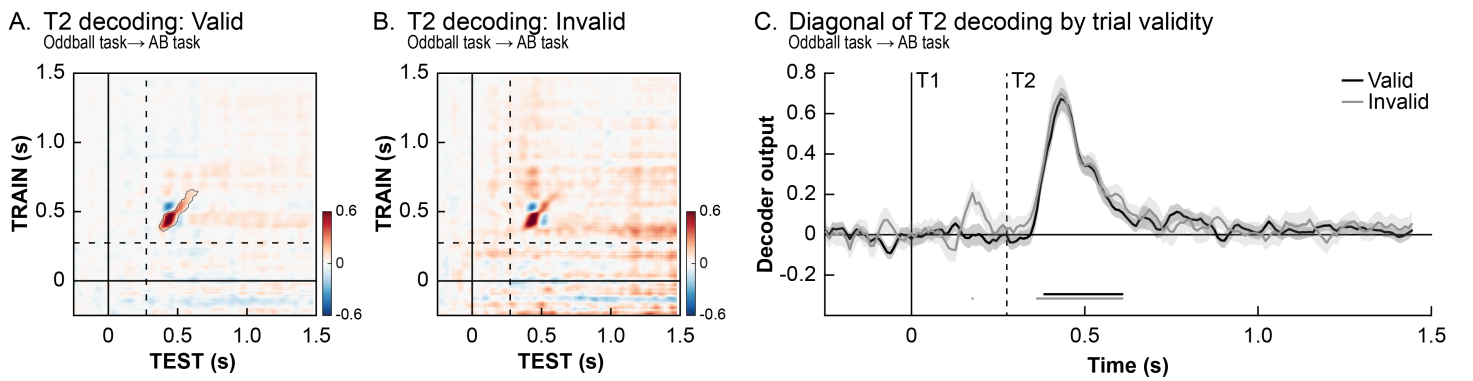
**

**Supplementary Figure 4. Effects of expectation validity on the neural representation of T2s when using data from all 28 subjects included in the behavioural analyses.** Temporal generalization matrices for the decoding of T2 identity at lag 3 for valid trials **(A)** and invalid trials **(B)** separately. Only trials where T1 was correctly identified were used. The pattern of results was similar to that in the main analyses reported in the Results section: there was no significant difference in the decoding of T2 identity between valid and invalid trials **(C).** Timing is relative to T1 onset, and the dashed lines indicate the onset of T2 stimuli at lag 3.

## ERF methods and results

In order to be able to make a more direct comparison with our earlier study (Meijs et al., 2018), we replicated the ERP analyses of main effects by performing two of ERF analyses in which we look at the effects of expectation validity and T2 visibility. Only lag 3 trials on which subject identified the correct T1 stimulus were used. Prior to the ERF analyses, the data was low-pass filtered at 40 Hz.

For each of the conditions in a comparison, the data were transformed to planar gradients to allow averaging over participants. Because this transformation is sensitive to the number of trials in a condition, we repeated this step 100 times for each condition, matching the number of trials between conditions that would be compared (by taking a random selection of trials from the largest condition) and averaging over these repetitions. Subsequently, we used cluster-based permutation tests (Maris & Oostenveld, 2007) on the time-window 750 ms after T2 onset to isolate significant ERF events relating to expectation validity (valid, invalid; irrespective of T2 visibility) or T2 visibility (seen, missed; irrespective of validity).

**Supplementary Figure 5** shows the results of the ERF analyses. In line with previous results (Meijs et al., 2018; Sergent et al., 2005), conscious detection of a target was reflected in a long-lasting P3-like component (p<0.001). However, we did not find an ERF effect analogous to the early negative posterior effect in the EEG results. Additionally, the comparison of the ERF on valid and invalid trials did not yield any significant differences (all p>0.332). Future research is required to explain the discrepancies in results between these studies, that may result from differences in task design or general differences between EEG and MEG.

**
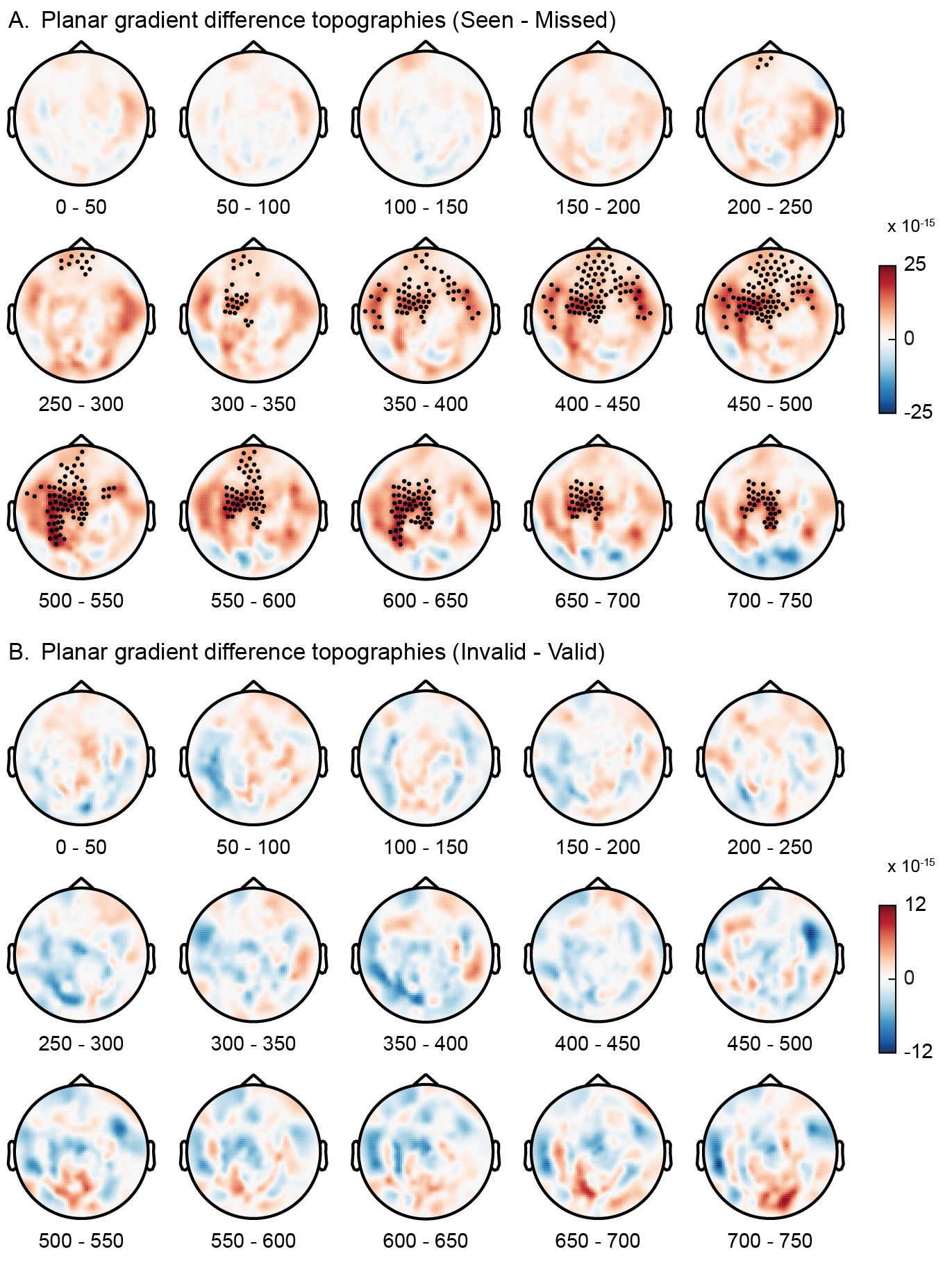
**

**Supplementary Figure 5. ERF results.** Topographic maps showing the difference between reported and missed T2s (**A)** and between valid and invalid expectations **(B)** over time (0 = *T2* onset) using planar gradients. Cluster-based permutation tests were used to isolate significant events. On every head map, channels belonging to a cluster that show significant difference for at least 50% of the time window are highlighted.

## Decoding expectation validation in the oddball task

Within the AB task we could not reliably decode whether a trial was valid or invalid. To find out whether effects of expectation were present in the data at all, we trained a classifier on the difference between standards and oddballs at lag 3 in the localizer task (trials with oddballs at other stimulus positions were not used for the analysis) and we then applied this to the AB task. This analysis may be sensitive to relatively general “mismatch” signals, potentially similar to mismatches in the AB task when comparing valid and invalid trials. As can be seen in **Supplementary Figure 6A**, no significant effects were observed when training on oddballs vs. standards in the oddball task and testing on invalid vs. valid trials in the AB task, suggesting an absence of a more general effect of expectation violation in the AB task (all p>0.495, note however that there are only a low number of oddball stimuli at lag 3 in the oddball task, around 20-25 per participant). However, within the oddball task, the difference between oddballs and standards could be reliably decoded (p<0.001; **Supplementary Figure 6B**).


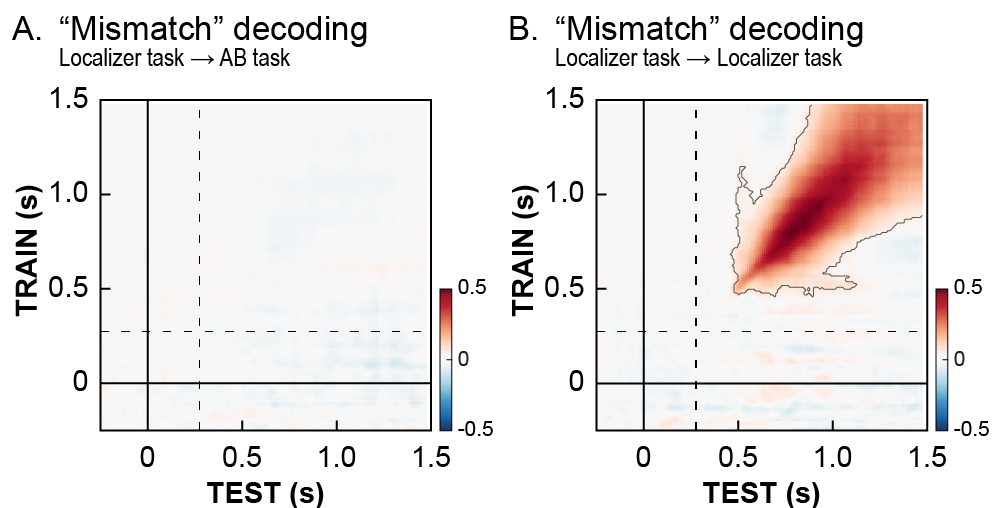


**Supplementary Figure 6. Decoding general effects of expectation by training a classifier on the difference between oddballs and standards (oddball task) and subsequently testing expectation violation within the AB task (A) and the oddball task itself (B). While a general mismatch effect could be decoded within the oddball task (using a 2-fold cross-validation procedure), no significant generalization to the AB task was observed.**
